# Supplementary material for: Clinical Characterization and Prognostic Value of TPM4 and Its Correlation with Epithelial–Mesenchymal Transition in Glioma
Source: Brain Sci. 2022 Aug 24;12(9):1120. doi: 10.3390/brainsci12091120 (PMC9497136; doi:10.3390/brainsci12091120)
Supplement: Supplementary file 1 [file brainsci-12-01120-s001.zip › Table_S1.pdf]

Supplemental Table 1 Patient characteristics in TCGA RNA-seq and CGGA_301 microarray data.

Characteristics	TCGA (n=697)	CGGA (n=301)
**Gender**		
male	370	180
female	271	121
NA	56	0
**Age (year)**	47 ± 15	42 ± 12
**TCGA Subtype**		
Classical	90	23
Mesenchymal	104	111
Proneural	248	86
Neural	115	81
NA	140	0
**WHO grade**		
Grade II	226	122
Grade III	249	51
Grade IV	167	128
NA	55	0
**KPS**	84 ± 14	NA
**IDH mutation status**		
Mutation	442	134
Wildtype	245	165
NA	10	2
**1p/19q Codeletion status**		
Codeletion	181	16
Non-codeletion	491	76
NA	25	209
**MGMT promoter status**		
Methylated	461	99
Unmethylated	162	187
NA	74	15

NA: Not Available; KPS: Karnofsky Performance Score; MGMT: O^6-Methylguanine Methyltransferase
