# Supplementary material for: Clinical Characterization and Prognostic Value of TPM4 and Its Correlation with Epithelial–Mesenchymal Transition in Glioma
Source: Brain Sci. 2022 Aug 24;12(9):1120. doi: 10.3390/brainsci12091120 (PMC9497136; doi:10.3390/brainsci12091120)
Supplement: Supplementary file 1 [file brainsci-12-01120-s001.zip › Table_S2.pdf]

Supplemental Table 2 Gene list for TPM4-significantly-correlated genes that overlap between CGGA and TCGA pan-glioma.

No.	Gene name
1	ABCC3
2	ACTA2
3	ACTG2
4	ACTN1
5	ADAM12
6	ADAM19
7	ADAM9
8	ADAMTS1
9	ADM
10	ADPGK
11	AFAP1L1
12	AK2
13	ANGPT2
14	ANPEP
15	ANXA1
16	ANXA2
17	ARAP3
18	ARHGAP11A
19	ARL4C

20	ARPC2
21	ARPC5
22	ASPM
23	ASPN
24	ATF3
25	ATF5
26	AURKA
27	BACE2
28	BCAT1
29	BCL10
30	BCL3
31	BDKRB2
32	BGN
33	BST1
34	BUB1
35	C11orf24
36	C15orf48
37	C1QTNF6
38	C1R
39	C1RL
40	CA9
41	CALD1

42	CALU
43	CANT1
44	CAPZA1
45	CASP4
46	CASP6
47	CASP8
48	CAV1
49	CCNB1
50	CCNB2
51	CD248
52	CD276
53	CD58
54	CD63
55	CD93
56	CDC6
57	CDCA2
58	CDCA8
59	CDKN3
60	CENPN
61	CEP55
62	CFI
63	CHEK2

64	CHI3L1
65	CHPF2
66	CHRNA9
67	CHSY1
68	CKAP4
69	CLCF1
70	CLIC1
71	CLIC4
72	CLSPN
73	CNIH4
74	COL18A1
75	COL1A1
76	COL1A2
77	COL3A1
78	COL4A1
79	COL4A2
80	COL5A1
81	COL5A2
82	COL6A1
83	COL6A2
84	COL8A1
85	CTHRC1

86	CTSB
87	CTSC
88	DCBLD2
89	DEPDC1
90	DLGAP5
91	DNAJB1
92	DNAJB11
93	DPYD
94	DUSP5
95	DUSP6
96	DYRK3
97	E2F7
98	E2F8
99	ECE1
100	ECSCR
101	EFNB2
102	EHD2
103	EHD4
104	ELF4
105	EMILIN1
106	EMILIN2
107	EMP3

108	ENG
109	ENPEP
110	EPHA2
111	ESM1
112	ETV6
113	FAM114A1
114	FAM126A
115	FAM20A
116	FAM20C
117	FBLIM1
118	FCGR2A
119	FCGR3A
120	FILIP1L
121	FKBP9
122	FLNA
123	FN1
124	FNDC3B
125	FOSL1
126	FOSL2
127	FSTL1
128	GADD45A
129	GAS2L3

130	GBP1
131	GDF15
132	GJC1
133	GLA
134	GLIPR1
135	GNG5
136	GNS
137	GPR4
138	GPX8
139	GUSB
140	HEXB
141	HIST1H2BH
142	HJURP
143	HK3
144	HSPA6
145	HSPG2
146	HTRA3
147	IBSP
148	ICAM1
149	IER5L
150	IFI30
151	IFNGR2

152	IGF2BP3
153	IGFBP2
154	IGFBP4
155	IKBIP
156	IL1RAP
157	IL2RA
158	IQGAP1
159	IQGAP2
160	ISG20
161	ITGA1
162	ITGA4
163	ITGA5
164	ITGB1
165	ITGB3
166	ITPRIPL1
167	JAG1
168	KCNE3
169	KDELR1
170	KDELR2
171	KDELR3
172	KIF20A
173	KIF23

174	KLF6
175	KYNU
176	LAMB1
177	LAMC1
178	LAMC3
179	LATS2
180	LDHA
181	LGALS1
182	LGALS3
183	LOX
184	LOXL1
185	LOXL2
186	LRRC32
187	LSP1
188	LUM
189	LXN
190	LYZ
191	MANF
192	MAP2K3
193	MCAM
194	METTL7B
195	MGP

196	MIR155HG
197	MMP11
198	MMP14
199	MMP19
200	MMP9
201	MPZL2
202	MPZL3
203	MSN
204	MSR1
205	MXRA5
206	MYL12A
207	MYL12B
208	MYL6
209	MYO1B
210	MYO1G
211	MYOF
212	NAMPT
213	NCAPG
214	NDUFA4L2
215	NEK6
216	NFKBIZ
217	NID2

218	NIP7
219	NNMT
220	NOX4
221	NRP1
222	NTAN1
223	OLFML2A
224	OSMR
225	OSTC
226	P4HA3
227	PCOLCE
228	PDIA3
229	PDIA5
230	PDLIM1
231	PDLIM7
232	PDPN
233	PECAM1
234	PFN1
235	PGM2
236	PHLDA2
237	PLAT
238	PLAU
239	PLAUR

240	PLBD1
241	PLEK2
242	PLEKHG2
243	PLIN2
244	PLK3
245	PLOD1
246	PLOD3
247	PLP2
248	PLSCR1
249	PLVAP
250	PMM2
251	POSTN
252	POTEF
253	PPP1R3B
254	PRF1
255	PROS1
256	PRSS23
257	PTGIR
258	PTTG1
259	PTX3
260	PYGL
261	RAB27A

262	RAB32
263	RAB42
264	RBMS1
265	RDH10
266	REXO2
267	RGS16
268	RGS3
269	RHOJ
270	RIPK1
271	RRM2
272	RUNX1
273	S100A11
274	S100A4
275	SAT1
276	SBNO2
277	SDC1
278	SEC24D
279	SECTM1
280	SEMA3F
281	SERPINB8
282	SERPINE1
283	SERPINH1

284	SERTAD1
285	SH2B3
286	SHC1
287	SHCBP1
288	SHISA5
289	SKA1
290	SLC10A3
291	SLC16A3
292	SLC25A24
293	SLC26A2
294	SLC30A7
295	SLC43A3
296	SMAGP
297	SMC4
298	SOCS3
299	SOD2
300	SPOCD1
301	SPON2
302	SPRY1
303	SRPX2
304	STEAP3
305	STK40

306	SUSD2
307	TACC3
308	TAGLN
309	TAGLN2
310	TEAD4
311	TES
312	TGFB1I1
313	TGFBI
314	TGIF1
315	THBD
316	THBS1
317	TIMP1
318	TK1
319	TM4SF1
320	TMEM45A
321	TMSB10
322	TMSB4X
323	TNC
324	TNFAIP6
325	TNFAIP8
326	TNFRSF11B
327	TNFRSF12A

328	TNFRSF1A
329	TPM4
330	TRAM2
331	TREM1
332	TUBA1C
333	TUBB6
334	TXNDC5
335	UGCG
336	VASP
337	VDR
338	VEGFA
339	VIM
340	VKORC1
341	WDR1
342	WEE1
343	ZWILCH
344	ZYX
