# Supplementary material for: Clinical Characterization and Prognostic Value of TPM4 and Its Correlation with Epithelial–Mesenchymal Transition in Glioma
Source: Brain Sci. 2022 Aug 24;12(9):1120. doi: 10.3390/brainsci12091120 (PMC9497136; doi:10.3390/brainsci12091120)
Supplement: Supplementary file 1 [file brainsci-12-01120-s001.zip › Table_S3.pdf]

Supplemental Table 3 Gene list for TPM4-significantly-correlated genes that overlap between CGGA and TCGA GBM

No.	Gene name
1	ACOT9
2	ACTA2
3	ACTN1
4	ADAM9
5	ADAMTS1
6	ARID5A
7	ARPC5
8	BACE2
9	BCL3
10	BGN
11	C1QTNF6
12	CALD1
13	CCDC102B
14	CD248
15	CD276
16	CD93
17	CHPF2
18	CLIC1
19	CNN2

20	COL12A1
21	COL18A1
22	COL4A1
23	COL4A2
24	COL5A1
25	COL5A2
26	COL6A1
27	COL6A2
28	DCBLD2
29	EIF4E2
30	ETV6
31	FAM20C
32	FBLIM1
33	FN1
34	FNDC3B
35	FOSL2
36	HIC1
37	HSPG2
38	IER5L
39	IGFBP4
40	IKBIP
41	ITGA1

42	ITGA5
43	ITGB1
44	ITGB3
45	LAMA4
46	LAMB1
47	LAMC1
48	LDHA
49	LMAN1
50	LMNA
51	LOXL1
52	LOXL2
53	LRRC32
54	MAFF
55	MAP2K3
56	MCAM
57	METRNL
58	MMP14
59	MPZL2
60	MYH9
61	MYL12A
62	MYL9
63	MYOF

64	NOX4
65	NRP1
66	P4HA2
67	PDGFRB
68	PDIA3
69	PDLIM7
70	PLAU
71	PLAUR
72	PPP1R3B
73	PRR16
74	RAB27A
75	RAI14
76	RBMS1
77	RCN3
78	RUNX1
79	SBNO2
80	SERPINB8
81	SERPINH1
82	TAGLN
83	TGFB1I1
84	TGFBI
85	THBD

86	THBS1
87	TPM2
88	TPM4
89	UACA
90	UBE3C
91	VASP
92	VCL
93	VIM
94	WWTR1
95	ZDHHC5
