# Supplementary material for: Clinical Characterization and Prognostic Value of TPM4 and Its Correlation with Epithelial–Mesenchymal Transition in Glioma
Source: Brain Sci. 2022 Aug 24;12(9):1120. doi: 10.3390/brainsci12091120 (PMC9497136; doi:10.3390/brainsci12091120)
Supplement: Supplementary file 1 [file brainsci-12-01120-s001.zip › Table_S4.pdf]

Supplemental Table 4 Gene sets of EMT-related signaling pathways

**Pathway**	**GeneID**
TGFB	TGFBR1
TGFB	SMAD7
TGFB	TGFB1
TGFB	SMURF2
TGFB	SMURF1
TGFB	BMPR2
TGFB	SKIL
TGFB	SKI
TGFB	ACVR1
TGFB	PMEPA1
TGFB	NCOR2
TGFB	SERPINE1
TGFB	JUNB
TGFB	SMAD1
TGFB	SMAD6
TGFB	PPP1R15A
TGFB	TGIF1
TGFB	FURIN
TGFB	SMAD3

TGFB	FKBP1A
TGFB	MAP3K7
TGFB	BMPR1A
TGFB	CTNNB1
TGFB	HIPK2
TGFB	KLF10
TGFB	BMP2
TGFB	ENG
TGFB	APC
TGFB	PPM1A
TGFB	XIAP
TGFB	CDH1
TGFB	ID1
TGFB	LEFTY2
TGFB	CDKN1C
TGFB	TRIM33
TGFB	RAB31
TGFB	TJP1
TGFB	SLC20A1
TGFB	CDK9
TGFB	ID3
TGFB	NOG

TGFB	ARID4B
TGFB	IFNGR2
TGFB	ID2
TGFB	PPP1CA
TGFB	SPTBN1
TGFB	WWTR1
TGFB	BCAR3
TGFB	THBS1
TGFB	FNTA
TGFB	HDAC1
TGFB	UBE2D3
TGFB	LTBP2
TGFB	RHOA
WNT	MYC
WNT	CTNNB1
WNT	JAG2
WNT	NOTCH1
WNT	DLL1
WNT	AXIN2
WNT	PSEN2
WNT	FZD1
WNT	NOTCH4

WNT	LEF1
WNT	AXIN1
WNT	NKD1
WNT	WNT5B
WNT	CUL1
WNT	JAG1
WNT	MAML1
WNT	KAT2A
WNT	GNAI1
WNT	WNT6
WNT	PTCH1
WNT	NCOR2
WNT	DKK4
WNT	HDAC2
WNT	DKK1
WNT	TCF7
WNT	WNT1
WNT	NUMB
WNT	ADAM17
WNT	DVL2
WNT	PPARD
WNT	NCSTN

WNT	HDAC5
WNT	CCND2
WNT	FRAT1
WNT	CSNK1E
WNT	RBPJ
WNT	FZD8
WNT	TP53
WNT	SKP2
WNT	HEY2
WNT	HEY1
WNT	HDAC11
PI3K_AKT	MAPK8
PI3K_AKT	PIK3R3
PI3K_AKT	GRB2
PI3K_AKT	NFKBIB
PI3K_AKT	MAP2K6
PI3K_AKT	MAPK9
PI3K_AKT	AKT1
PI3K_AKT	MAPK1
PI3K_AKT	PLCG1
PI3K_AKT	TRIB3
PI3K_AKT	GSK3B

PI3K_AKT	MAP2K3
PI3K_AKT	CDKN1A
PI3K_AKT	RAC1
PI3K_AKT	RIPK1
PI3K_AKT	AKT1S1
PI3K_AKT	ACTR2
PI3K_AKT	PRKAR2A
PI3K_AKT	YWHAB
PI3K_AKT	HRAS
PI3K_AKT	PDK1
PI3K_AKT	PIKFYVE
PI3K_AKT	TBK1
PI3K_AKT	ACTR3
PI3K_AKT	E2F1
PI3K_AKT	MYD88
PI3K_AKT	ITPR2
PI3K_AKT	SQSTM1
PI3K_AKT	RPS6KA1
PI3K_AKT	PTPN11
PI3K_AKT	MAPKAP1
PI3K_AKT	PLCB1
PI3K_AKT	RAF1

PI3K_AKT	CAMK4
PI3K_AKT	RPTOR
PI3K_AKT	CFL1
PI3K_AKT	CDK4
PI3K_AKT	TRAF2
PI3K_AKT	GNGT1
PI3K_AKT	UBE2N
PI3K_AKT	ADCY2
PI3K_AKT	CDKN1B
PI3K_AKT	VAV3
PI3K_AKT	FGF6
PI3K_AKT	ECSIT
PI3K_AKT	RALB
PI3K_AKT	ARF1
PI3K_AKT	MKNK1
PI3K_AKT	CDK1
PI3K_AKT	PTEN
PI3K_AKT	ARHGDIA
PI3K_AKT	GRK2
PI3K_AKT	FGF17
PI3K_AKT	DDIT3
PI3K_AKT	AC093012.1

PI3K_AKT	TIAM1
PI3K_AKT	CDK2
PI3K_AKT	SFN
PI3K_AKT	PRKCB
PI3K_AKT	GNA14
PI3K_AKT	EIF4E
PI3K_AKT	CLTC
PI3K_AKT	TSC2
PI3K_AKT	FGF22
PI3K_AKT	PPP1CA
PI3K_AKT	DUSP3
PI3K_AKT	HSP90B1
PI3K_AKT	IL4
PI3K_AKT	STAT2
PI3K_AKT	SLA
PI3K_AKT	EGFR
PI3K_AKT	PLA2G12A
PI3K_AKT	MAPK10
PI3K_AKT	CALR
PI3K_AKT	THEM4
PI3K_AKT	RIT1
PI3K_AKT	MKNK2

PI3K_AKT	PPP2R1B
PI3K_AKT	CAB39L
PI3K_AKT	ARPC3
PI3K_AKT	PITX2
PI3K_AKT	NCK1
PI3K_AKT	IL2RG
PI3K_AKT	PFN1
PI3K_AKT	FASLG
PI3K_AKT	NOD1
PI3K_AKT	DAPP1
PI3K_AKT	UBE2D3
PI3K_AKT	CAB39
PI3K_AKT	AP2M1
PI3K_AKT	MAP3K7
PI3K_AKT	PRKAG1
PI3K_AKT	CSNK2B
PI3K_AKT	PRKAA2
PI3K_AKT	ATF1
PI3K_AKT	SLC2A1
PI3K_AKT	PIN1
PI3K_AKT	TNFRSF1A
PI3K_AKT	LCK

PI3K_AKT	RPS6KA3
PI3K_AKT	NGF
PI3K_AKT	CXCR4
PI3K_AKT	ACACA
PI3K_AKT	SMAD2
PI3K_AKT	PAK4
MAPK	AKT1
MAPK	AKT2
MAPK	AKT3
MAPK	ARRB1
MAPK	ARRB2
MAPK	ATF2
MAPK	ATF4
MAPK	BDNF
MAPK	BRAF
MAPK	CACNA1A
MAPK	CACNA1B
MAPK	CACNA1C
MAPK	CACNA1D
MAPK	CACNA1E
MAPK	CACNA1F
MAPK	CACNA1G

MAPK	CACNA1H
MAPK	CACNA1I
MAPK	CACNA1S
MAPK	CACNA2D1
MAPK	CACNA2D2
MAPK	CACNA2D3
MAPK	CACNA2D4
MAPK	CACNB1
MAPK	CACNB2
MAPK	CACNB3
MAPK	CACNB4
MAPK	CACNG1
MAPK	CACNG2
MAPK	CACNG3
MAPK	CACNG4
MAPK	CACNG5
MAPK	CACNG6
MAPK	CACNG7
MAPK	CACNG8
MAPK	CASP3
MAPK	CD14
MAPK	CDC25B

MAPK	CDC42
MAPK	CHP1
MAPK	CHP2
MAPK	CHUK
MAPK	CRK
MAPK	CRKL
MAPK	DAXX
MAPK	DDIT3
MAPK	DUSP1
MAPK	DUSP10
MAPK	DUSP14
MAPK	DUSP16
MAPK	DUSP2
MAPK	DUSP3
MAPK	DUSP4
MAPK	DUSP5
MAPK	DUSP6
MAPK	DUSP7
MAPK	DUSP8
MAPK	DUSP9
MAPK	ECSIT
MAPK	EGF

MAPK	EGFR
MAPK	ELK1
MAPK	ELK4
MAPK	FAS
MAPK	FASLG
MAPK	FGF1
MAPK	FGF10
MAPK	FGF11
MAPK	FGF12
MAPK	FGF13
MAPK	FGF14
MAPK	FGF16
MAPK	FGF17
MAPK	FGF18
MAPK	FGF19
MAPK	FGF2
MAPK	FGF20
MAPK	FGF21
MAPK	FGF22
MAPK	FGF23
MAPK	FGF3
MAPK	FGF4

MAPK	FGF5
MAPK	FGF6
MAPK	FGF7
MAPK	FGF8
MAPK	FGF9
MAPK	FGFR1
MAPK	FGFR2
MAPK	FGFR3
MAPK	FGFR4
MAPK	FLNA
MAPK	FLNB
MAPK	FLNC
MAPK	FOS
MAPK	GADD45A
MAPK	GADD45B
MAPK	GADD45G
MAPK	GNA12
MAPK	GNG12
MAPK	GRB2
MAPK	HRAS
MAPK	HSPA1A
MAPK	HSPA1B

MAPK	HSPA1L
MAPK	HSPA2
MAPK	HSPA6
MAPK	HSPA8
MAPK	HSPB1
MAPK	IKBKB
MAPK	IKBKG
MAPK	IL1A
MAPK	IL1B
MAPK	IL1R1
MAPK	IL1R2
MAPK	JMJD7-PLA2G4B
MAPK	JUN
MAPK	JUND
MAPK	KRAS
MAPK	LAMTOR3
MAPK	MAP2K1
MAPK	MAP2K2
MAPK	MAP2K3
MAPK	MAP2K4
MAPK	MAP2K5
MAPK	MAP2K6

MAPK	MAP2K7
MAPK	MAP3K1
MAPK	MAP3K11
MAPK	MAP3K12
MAPK	MAP3K13
MAPK	MAP3K14
MAPK	MAP3K2
MAPK	MAP3K20
MAPK	MAP3K3
MAPK	MAP3K4
MAPK	MAP3K5
MAPK	MAP3K6
MAPK	MAP3K7
MAPK	MAP3K8
MAPK	MAP4K1
MAPK	MAP4K2
MAPK	MAP4K3
MAPK	MAP4K4
MAPK	MAPK1
MAPK	MAPK10
MAPK	MAPK11
MAPK	MAPK12

MAPK	MAPK13
MAPK	MAPK14
MAPK	MAPK3
MAPK	MAPK7
MAPK	MAPK8
MAPK	MAPK8IP1
MAPK	MAPK8IP2
MAPK	MAPK8IP3
MAPK	MAPK9
MAPK	MAPKAPK2
MAPK	MAPKAPK3
MAPK	MAPKAPK5
MAPK	MAPT
MAPK	MAX
MAPK	MECOM
MAPK	MEF2C
MAPK	MKNK1
MAPK	MKNK2
MAPK	MOS
MAPK	MRAS
MAPK	MYC
MAPK	NF1

MAPK	NFATC2
MAPK	NFATC4
MAPK	NFKB1
MAPK	NFKB2
MAPK	NGF
MAPK	NLK
MAPK	NR4A1
MAPK	NRAS
MAPK	NTF3
MAPK	NTF4
MAPK	NTRK1
MAPK	NTRK2
MAPK	PAK1
MAPK	PAK2
MAPK	PDGFA
MAPK	PDGFB
MAPK	PDGFRA
MAPK	PDGFRB
MAPK	PLA2G10
MAPK	PLA2G12A
MAPK	PLA2G12B
MAPK	PLA2G1B

MAPK	PLA2G2A
MAPK	PLA2G2C
MAPK	PLA2G2D
MAPK	PLA2G2E
MAPK	PLA2G2F
MAPK	PLA2G3
MAPK	PLA2G4A
MAPK	PLA2G4B
MAPK	PLA2G4E
MAPK	PLA2G5
MAPK	PLA2G6
MAPK	PPM1A
MAPK	PPM1B
MAPK	PPP3CA
MAPK	PPP3CB
MAPK	PPP3CC
MAPK	PPP3R1
MAPK	PPP3R2
MAPK	PPP5C
MAPK	PRKACA
MAPK	PRKACB
MAPK	PRKACG

MAPK	PRKCA
MAPK	PRKCB
MAPK	PRKCG
MAPK	PRKX
MAPK	PTPN5
MAPK	PTPN7
MAPK	PTPRR
MAPK	RAC1
MAPK	RAC2
MAPK	RAC3
MAPK	RAF1
MAPK	RAP1A
MAPK	RAP1B
MAPK	RAPGEF2
MAPK	RASA1
MAPK	RASA2
MAPK	RASGRF1
MAPK	RASGRF2
MAPK	RASGRP1
MAPK	RASGRP2
MAPK	RASGRP3
MAPK	RASGRP4

MAPK	RELA
MAPK	RELB
MAPK	RPS6KA1
MAPK	RPS6KA2
MAPK	RPS6KA3
MAPK	RPS6KA4
MAPK	RPS6KA5
MAPK	RPS6KA6
MAPK	RRAS
MAPK	RRAS2
MAPK	SOS1
MAPK	SOS2
MAPK	SRF
MAPK	STK3
MAPK	STK4
MAPK	STMN1
MAPK	TAB1
MAPK	TAB2
MAPK	TAOK1
MAPK	TAOK2
MAPK	TAOK3
MAPK	TGFB1

MAPK	TGFB2
MAPK	TGFB3
MAPK	TGFBR1
MAPK	TGFBR2
MAPK	TNF
MAPK	TNFRSF1A
MAPK	TP53
MAPK	TRAF2
MAPK	TRAF6
Notch	JAG1
Notch	NOTCH3
Notch	NOTCH2
Notch	APH1A
Notch	HES1
Notch	CCND1
Notch	FZD1
Notch	PSEN2
Notch	FZD7
Notch	DTX1
Notch	DLL1
Notch	FZD5
Notch	MAML2

Notch	NOTCH1
Notch	PSENEN
Notch	WNT5A
Notch	CUL1
Notch	WNT2
Notch	DTX4
Notch	SAP30
Notch	PPARD
Notch	KAT2A
Notch	HEYL
Notch	SKP1
Notch	RBX1
Notch	TCF7L2
Notch	ARRB1
Notch	LFNG
Notch	PRKCA
Notch	DTX2
Notch	ST3GAL6
Notch	FBXW11
Hedgehog	SHH
Hedgehog	PTCH1
Hedgehog	NRCAM

Hedgehog	NRP1
Hedgehog	SCG2
Hedgehog	AMOT
Hedgehog	UNC5C
Hedgehog	ADGRG1
Hedgehog	HEY1
Hedgehog	GLI1
Hedgehog	THY1
Hedgehog	SLIT1
Hedgehog	CDK6
Hedgehog	HEY2
Hedgehog	NRP2
Hedgehog	TLE3
Hedgehog	TLE1
Hedgehog	L1CAM
Hedgehog	PLG
Hedgehog	NKX6-1
Hedgehog	NF1
Hedgehog	RASA1
Hedgehog	ETS2
Hedgehog	RTN1
Hedgehog	CRMP1

Hedgehog	MYH9
Hedgehog	VEGFA
Hedgehog	CELSR1
Hedgehog	CNTFR
Hedgehog	ACHE
Hedgehog	PML
Hedgehog	CDK5R1
Hedgehog	VLDLR
Hedgehog	OPHN1
Hedgehog	LDB1
Hedgehog	DPYSL2
Hypoxia	PGK1
Hypoxia	PDK1
Hypoxia	GBE1
Hypoxia	PFKL
Hypoxia	ALDOA
Hypoxia	ENO2
Hypoxia	PGM1
Hypoxia	NDRG1
Hypoxia	HK2
Hypoxia	ALDOC
Hypoxia	GPI

Hypoxia	MXI1
Hypoxia	SLC2A1
Hypoxia	P4HA1
Hypoxia	ADM
Hypoxia	P4HA2
Hypoxia	ENO1
Hypoxia	PFKP
Hypoxia	AK4
Hypoxia	FAM162A
Hypoxia	PFKFB3
Hypoxia	VEGFA
Hypoxia	BNIP3L
Hypoxia	TPI1
Hypoxia	ERO1A
Hypoxia	KDM3A
Hypoxia	CCNG2
Hypoxia	LDHA
Hypoxia	GYS1
Hypoxia	GAPDH
Hypoxia	BHLHE40
Hypoxia	ANGPTL4
Hypoxia	JUN

Hypoxia	SERPINE1
Hypoxia	LOX
Hypoxia	GCK
Hypoxia	PPFIA4
Hypoxia	MAFF
Hypoxia	DDIT4
Hypoxia	SLC2A3
Hypoxia	IGFBP3
Hypoxia	NFIL3
Hypoxia	FOS
Hypoxia	RBPJ
Hypoxia	HK1
Hypoxia	CITED2
Hypoxia	ISG20
Hypoxia	GALK1
Hypoxia	WSB1
Hypoxia	PYGM
Hypoxia	STC1
Hypoxia	ZNF292
Hypoxia	BTG1
Hypoxia	PLIN2
Hypoxia	CSRP2

Hypoxia	VLDLR
Hypoxia	JMJD6
Hypoxia	EXT1
Hypoxia	F3
Hypoxia	PDK3
Hypoxia	ANKZF1
Hypoxia	UGP2
Hypoxia	ALDOB
Hypoxia	STC2
Hypoxia	ERRFI1
Hypoxia	ENO3
Hypoxia	PNRC1
Hypoxia	HMOX1
Hypoxia	PGF
Hypoxia	GAPDHS
Hypoxia	CHST2
Hypoxia	TMEM45A
Hypoxia	BCAN
Hypoxia	ATF3
Hypoxia	CAV1
Hypoxia	AMPD3
Hypoxia	GPC3

Hypoxia	NDST1
Hypoxia	IRS2
Hypoxia	SAP30
Hypoxia	GAA
Hypoxia	SDC4
Hypoxia	STBD1
Hypoxia	IER3
Hypoxia	PKLR
Hypoxia	IGFBP1
Hypoxia	PLAUR
Hypoxia	CAVIN3
Hypoxia	CCN5
Hypoxia	LARGE1
Hypoxia	NOCT
Hypoxia	S100A4
Hypoxia	RRAGD
Hypoxia	ZFP36
Hypoxia	EGFR
Hypoxia	EDN2
Hypoxia	IDS
Hypoxia	CDKN1A
Hypoxia	RORA

Hypoxia	DUSP1
Hypoxia	MIF
Hypoxia	PPP1R3C
Hypoxia	DPYSL4
Hypoxia	KDELR3
Hypoxia	DTNA
Hypoxia	ADORA2B
Hypoxia	HS3ST1
Hypoxia	CAVIN1
Hypoxia	NR3C1
Hypoxia	KLF6
Hypoxia	GPC4
Hypoxia	CCN1
Hypoxia	TNFAIP3
Hypoxia	CA12
Hypoxia	HEXA
Hypoxia	BGN
Hypoxia	PPP1R15A
Hypoxia	PGM2
Hypoxia	PIM1
Hypoxia	PRDX5
Hypoxia	NAGK

Hypoxia	CDKN1B
Hypoxia	BRS3
Hypoxia	TKTL1
Hypoxia	MT1E
Hypoxia	ATP7A
Hypoxia	MT2A
Hypoxia	SDC3
Hypoxia	TIPARP
Hypoxia	PKP1
Hypoxia	ANXA2
Hypoxia	PGAM2
Hypoxia	DDIT3
Hypoxia	PRKCA
Hypoxia	SLC37A4
Hypoxia	CXCR4
Hypoxia	EFNA3
Hypoxia	CP
Hypoxia	KLF7
Hypoxia	CCN2
Hypoxia	CHST3
Hypoxia	TPD52
Hypoxia	LXN

Hypoxia	B4GALNT2
Hypoxia	PPARGC1A
Hypoxia	BCL2
Hypoxia	GCNT2
Hypoxia	HAS1
Hypoxia	KLHL24
Hypoxia	SCARB1
Hypoxia	SLC25A1
Hypoxia	SDC2
Hypoxia	CASP6
Hypoxia	VHL
Hypoxia	FOXO3
Hypoxia	PDGFB
Hypoxia	B3GALT6
Hypoxia	SLC2A5
Hypoxia	SRPX
Hypoxia	EFNA1
Hypoxia	GLRX
Hypoxia	ACKR3
Hypoxia	PAM
Hypoxia	TGFBI
Hypoxia	DCN

Hypoxia	SIAH2
Hypoxia	PLAC8
Hypoxia	FBP1
Hypoxia	TPST2
Hypoxia	PHKG1
Hypoxia	MYH9
Hypoxia	CDKN1C
Hypoxia	GRHPR
Hypoxia	PCK1
Hypoxia	INHA
Hypoxia	HSPA5
Hypoxia	NDST2
Hypoxia	NEDD4L
Hypoxia	TPBG
Hypoxia	XPNPEP1
Hypoxia	IL6
Hypoxia	SLC6A6
Hypoxia	MAP3K1
Hypoxia	LDHC
Hypoxia	AKAP12
Hypoxia	TES
Hypoxia	KIF5A

Hypoxia	LALBA
Hypoxia	COL5A1
Hypoxia	GPC1
Hypoxia	HDLBP
Hypoxia	ILVBL
Hypoxia	NCAN
Hypoxia	TGM2
Hypoxia	ETS1
Hypoxia	HOXB9
Hypoxia	SELENBP1
Hypoxia	FOSL2
Hypoxia	SULT2B1
Hypoxia	TGFB3
