# Supplementary figures and images for: Clinical Characterization and Prognostic Value of TPM4 and Its Correlation with Epithelial–Mesenchymal Transition in Glioma
Source: Brain Sci. 2022 Aug 24;12(9):1120. doi: 10.3390/brainsci12091120 (PMC9497136; doi:10.3390/brainsci12091120)

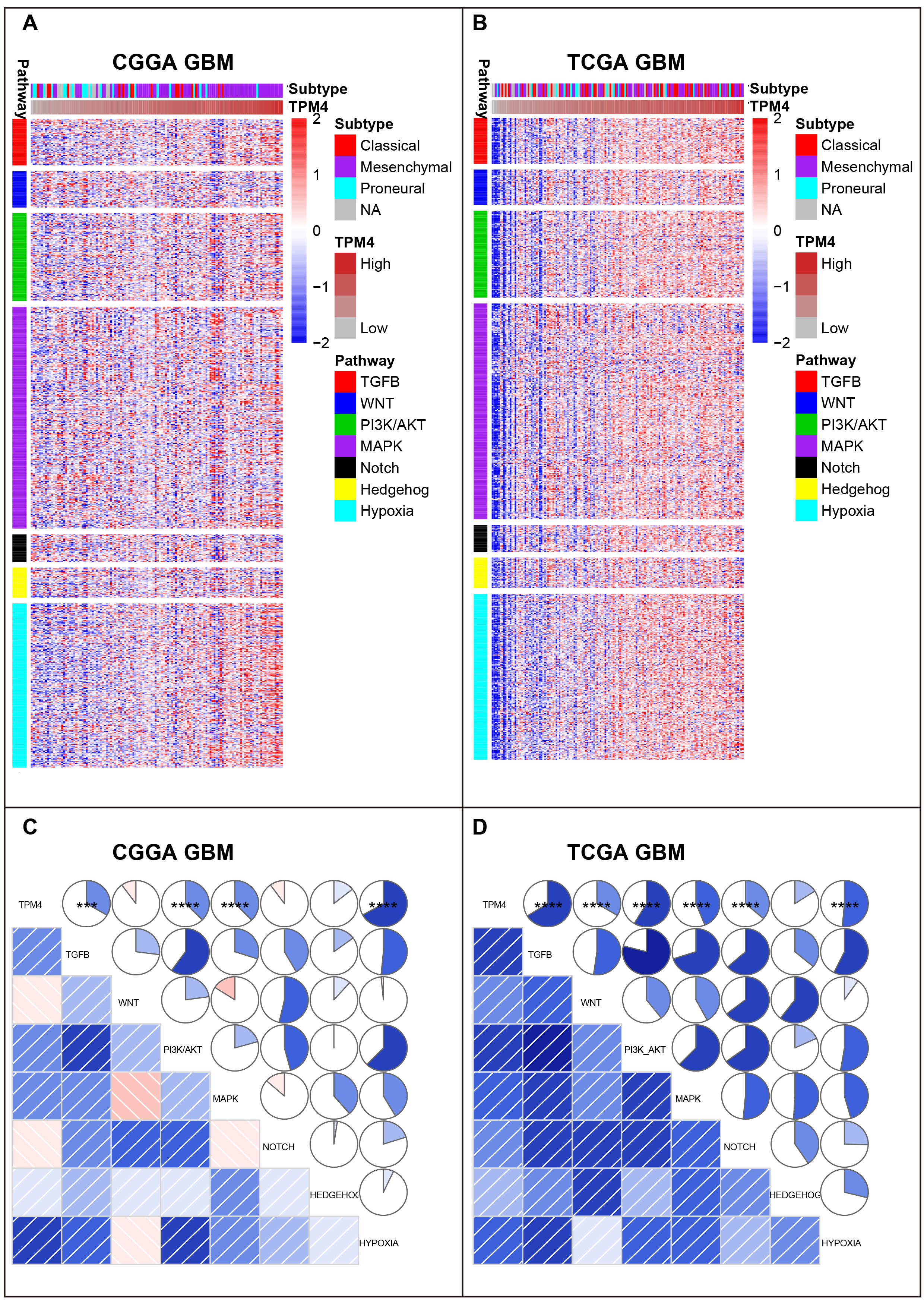

Supplement: Supplementary file 1 [file brainsci-12-01120-s001.zip › Figure_S1.tif]

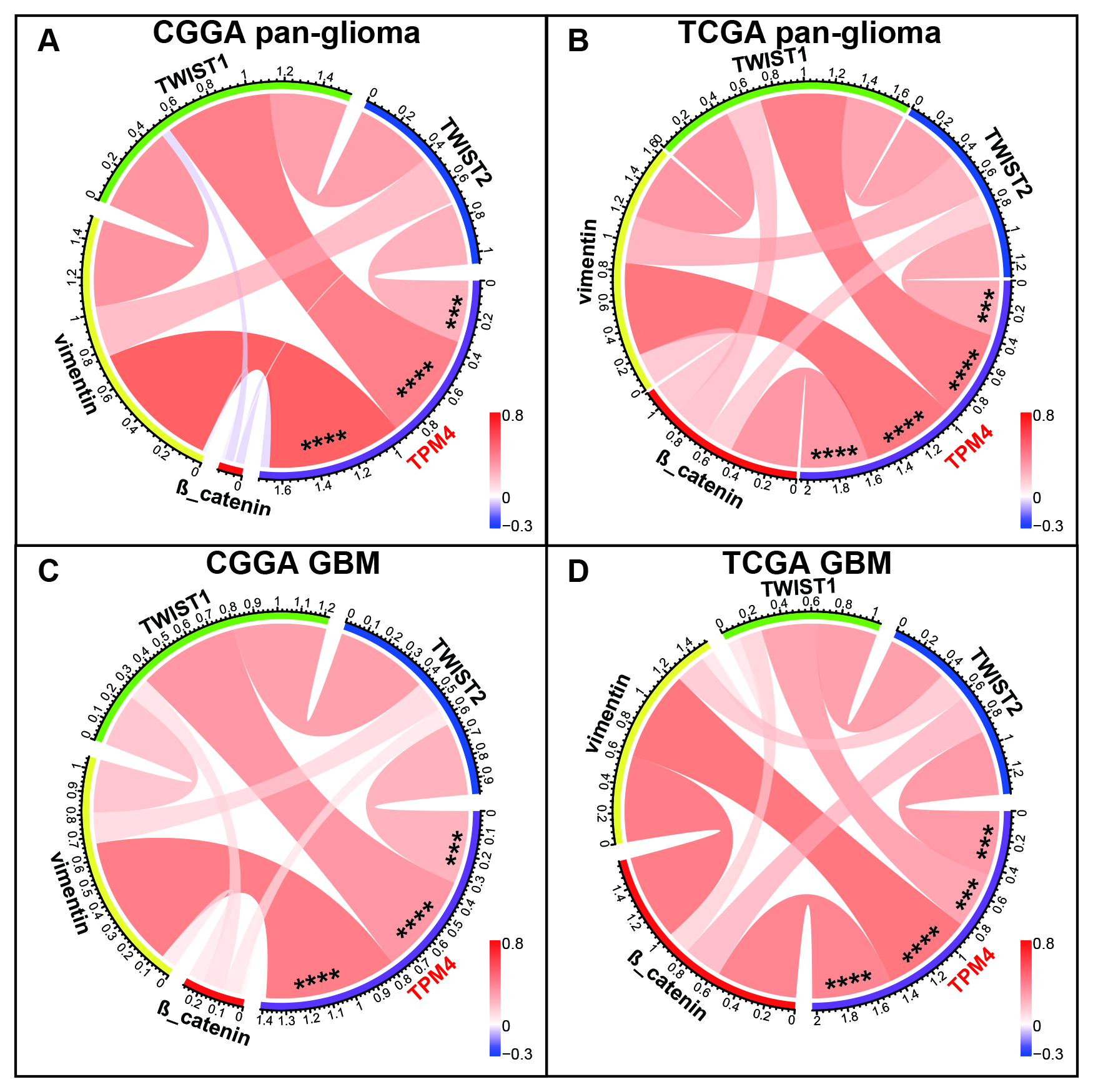

Supplement: Supplementary file 1 [file brainsci-12-01120-s001.zip › Figure_S2.tif]

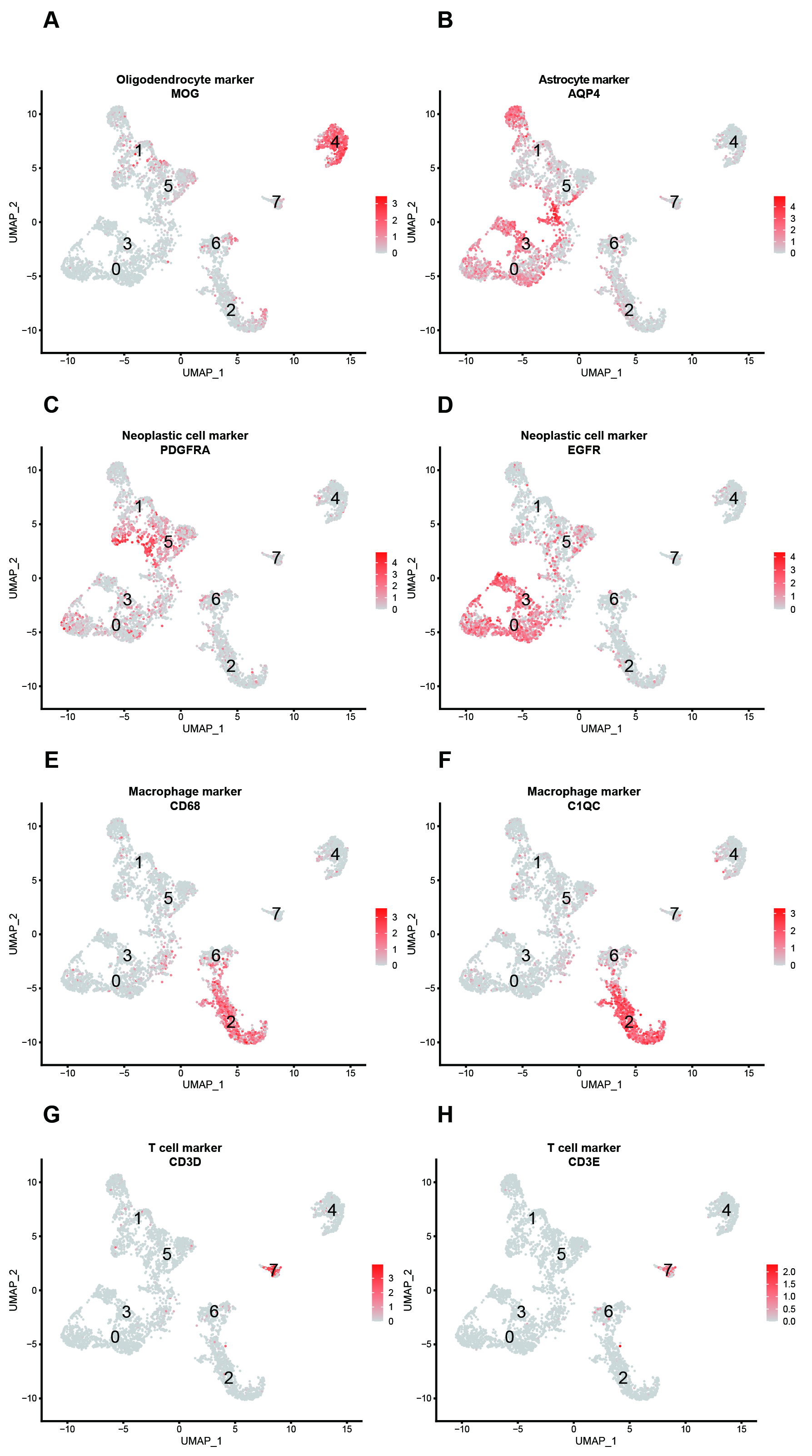

Supplement: Supplementary file 1 [file brainsci-12-01120-s001.zip › Figure_S3.tif]

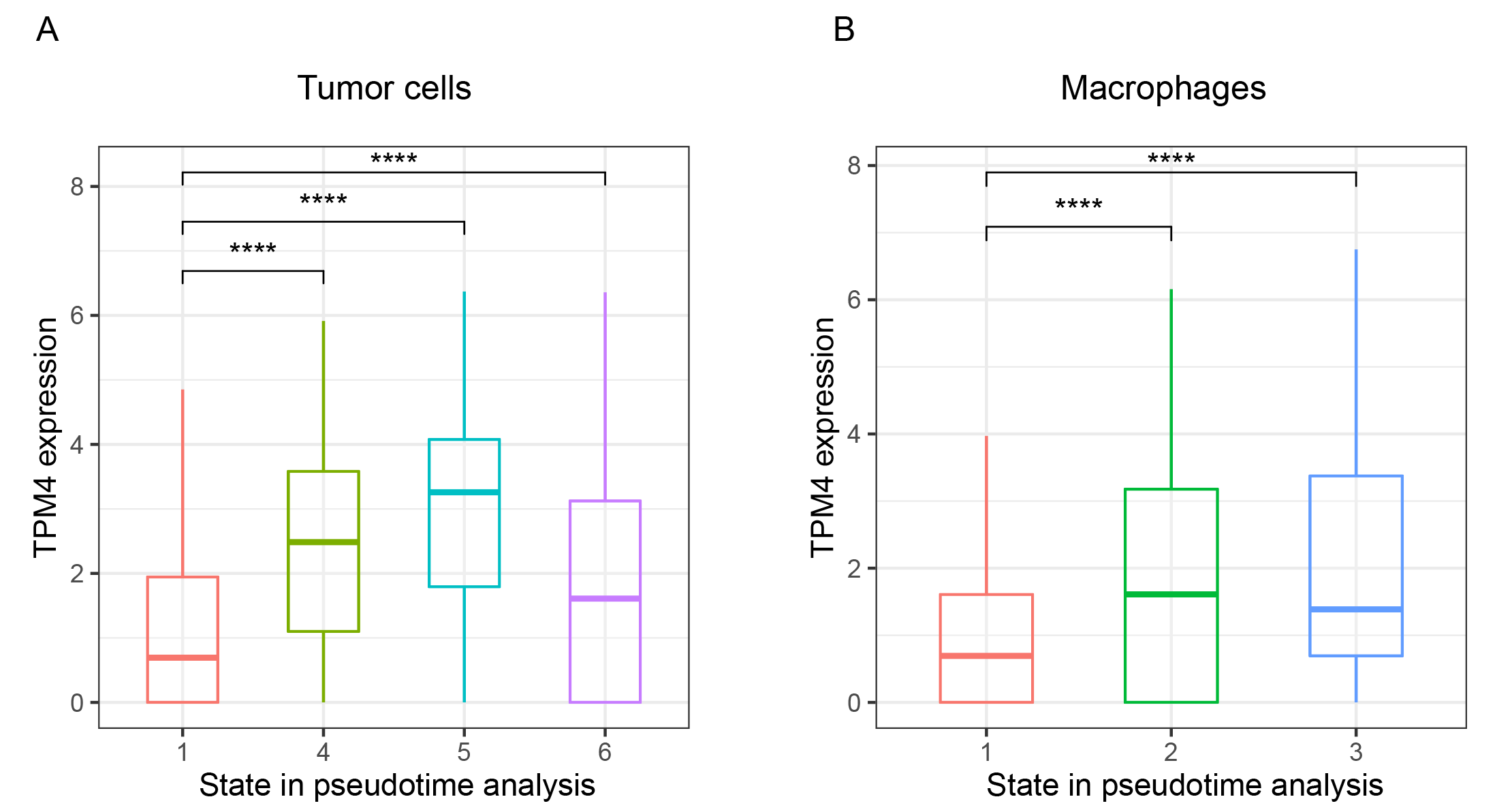

Supplement: Supplementary file 1 [file brainsci-12-01120-s001.zip › Figure_S4.tif]
